# Encapsulation of macrophages enhances their retention and angiogenic potential

**DOI:** 10.1038/s41536-019-0068-5

**Published:** 2019-03-20

**Authors:** Francesca E. Ludwinski, Ashish S. Patel, Gopinath Damodaran, Jun Cho, Joanna Furmston, Qingbo Xu, Suwan N. Jayasinghe, Alberto Smith, Bijan Modarai

**Affiliations:** 1 King’s College London, Academic Department of Vascular Surgery, School of Cardiovascular Medicine & Sciences, BHF Centre for Regenerative Medicine and BHF Centre of Excellence and the Biomedical Research Centre at Guy’s & St Thomas’ NHS Foundation Trust and King’s College London, London, UK; 2 King’s College London, Vascular Biology Section, School of Cardiovascular Medicine & Sciences, BHF Centre of Excellence, King’s College London, London, UK; 3 BioPhysics Group, UCL Centre for Stem Cells and Regenerative Medicine, UCL Department of Mechanical Engineering and UCL Institute of Healthcare Engineering, University College London, Torrington Place, London, WC1E 7JE UK

## Abstract

Cell therapies to treat critical limb ischaemia have demonstrated only modest results in clinical trials, and this has been partly attributed to poor cell retention following their delivery directly into the ischaemic limb. The aim of this study was to determine whether alginate encapsulation of therapeutic pro-angio/arteriogenic macrophages enhances their retention and ultimately improves limb perfusion. A reproducible GMP-compliant method for generating 300 µm alginate capsules was developed to encapsulate pro-angio/arteriogenic macrophages. Longitudinal analysis revealed no detrimental effect of encapsulation on cell number or viability in vitro, and macrophages retained their pro-angio/arteriogenic phenotype. Intramuscular delivery of encapsulated macrophages into the murine ischaemic hindlimb demonstrated increased cell retention compared with injection of naked cells (*P* = 0.0001), and that this was associated both enhanced angiogenesis (*P* = 0.02) and arteriogenesis (*P* = 0.03), and an overall improvement in limb perfusion (*P* = 0.0001). Alginate encapsulation of pro-angio/arteriogenic macrophages enhances cell retention and subsequent limb reperfusion in vivo. Encapsulation may therefore represent a means of improving the efficacy of cell-based therapies currently under investigation for the treatment of limb ischaemia.

## Limb ischemia: Encapsulation enhances therapeutic promise of immune cells in mice

Blood vessel-promoting immune cells stay longer in the body and help promote blood flow to the feet and toes of mice with critical limb ischemia when the therapeutic cells are packaged inside tiny bubbles of a biocompatible seaweed derivative called alginate. A team led by Bijan Modarai from King’s College London, UK, developed a reliable method for placing artery-stimulating macrophage cells inside alginate capsules measuring 300 micrometres in diameter, about the thickness of a postcard. In culture, the alginate coating had no effect on the macrophage viability; and when injected into the muscles of mice with artery blockages to their hindlimbs, the encapsulated cells were retained longer and offered greater therapeutic benefit than uncoated cells. This encapsulation strategy may improve the efficacy of comparable cell-based therapies for humans with limb ischemia.

## Introduction

Critical limb ischaemia (CLI) is a severe manifestation of peripheral arterial disease (PAD) that is characterised by pain and gangrene.^1^ The limb salvage rate for patients with CLI remains poor,^2^ with a significant proportion of patients not amenable to standard treatments, including surgical bypass and angioplasty. This has been the driver for the development of angiogenic cell-based therapies aimed at limb salvage in these no option patients. Clinical trials of cell therapy to date have only shown a modest benefit with disappointing results attributed to the lack of potency of cells injected, including a functional impairment of autologous cells harvested from patients with multiple co-morbidities.^3–10^

The poor retention of cells after injection into the target site is also thought to limit their potential for effecting robust collateralisation. Cells injected directly into the calf muscle are susceptible to clearance by immune cells or apoptosis triggered by the hypoxic, pro-inflammatory environment.^11^ Mononuclear cells injected intramuscularly in the ischaemic hindlimb have a short-lived survival, which is not improved with repeated injection.^12^ There are currently no studies to assess retention of cells injected into the ischaemic limb in man, but clinical studies of therapeutic cell injection into the heart reveal a similar precipitous loss, with only ~12% of cells retained after 1 h.^13^

The use of implantable biomaterials, containing therapeutic cells, to enhance cell-based therapies is gaining traction in a number of cell therapy areas, including the use of bone marrow-derived mesenchymal stem cells for revascularisation of infarcted myocardium and ischaemic hindlimbs.^14,15^ Encapsulation in polymeric matrices, including alginate, can be used to deliver therapeutic cells as it not only enhances cellular retention and survival,^16,17^ but also provides a semi-permeable membrane for diffusion of nutrients, stimulants and waste products.^18^

Alginate is an unbranched algae-derived polysaccharide, which gels upon contact with divalent cations.^19^ Its biocompatibility, paired with ease of use makes it an attractive option for the development of cell therapy. We have previously identified a subset of human monocyte/macrophages that promote limb revascularisation in mice^20^ and carried out a first in man study involving delivery of this subset in patients with limb ischaemia (unpublished data). Here, we use murine pro-angio/arteriogenic macrophages to optimise and standardise a good manufacturing practice (GMP)-compliant encapsulation strategy, and to study the effect of this procedure on their viability and capacity to enhance revascularisation of the ischaemic limb, in readiness for clinical trials in patients with limb ischaemia.

## Results

### Optimisation of alginate capsule generation

**Fig. 1:**
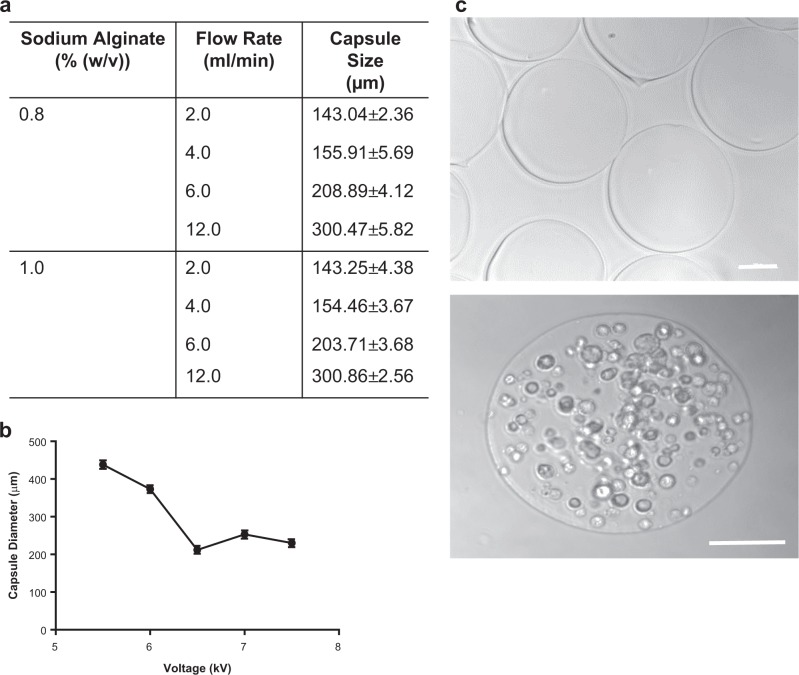
Optimisation of capsule generation. **a** The diameter of alginate capsules generated under a range of alginate concentrations (0.8% and 1.0%), and encapsulator flow rates (2–12 ml/min) was measured microscopically (*n* = 10/group). **b** Variation of the encapsulator voltage settings influenced the diameter of capsules generated (*n* = 5/group, error bars = s.d.). **c** Consistently round 300 µm diameter capsules were generated using 1.0% alginate with a flow rate of 12 ml/min and were used to encapsulate Tie2-iBMMs at a cell density of 1 × 10^7^ cells/ml. Scale bar = 100 µm

A number of encapsulation parameters were optimised to allow reproducible generation of capsules of a consistent shape and diameter, prior to generating cell-seeded capsules for subsequent in vitro and in vivo experiments. Capsule diameter was affected by increasing the flow rate of alginate solution through the cell encapsulator, but varying the concentration of sodium alginate had little effect (Fig. 1a). Increasing the voltage applied to the alginate suspension decreased the capsule diameter (Fig. 1b). A flow rate of 12 ml/min, with 1.0% sodium alginate and 6.8 kV reliably produced capsules of 300 µm diameter and a round shape (Fig. 1a, c).

In order to standardise experimental conditions, we encapsulated immortalised murine bone marrow-derived macrophages engineered to express the Tie2 receptor (Tie2-iBMMs, see Methods) to provide a uniform population of angiogenic cells for these proof-of-concept studies aimed at developing a standardised GMP-compliant encapsulation method and deciphering the effect of encapsulation on cells. Uniformly seeded capsules were produced when Tie2-iBMMs were seeded into the alginate solution at a concentration of 1 × 10^7^ cells/ml, with capsules containing approximately 200 cells each (Fig. 1c).

### The effect of encapsulation on Tie2-iBMM viability and phenotype

**Fig. 2:**
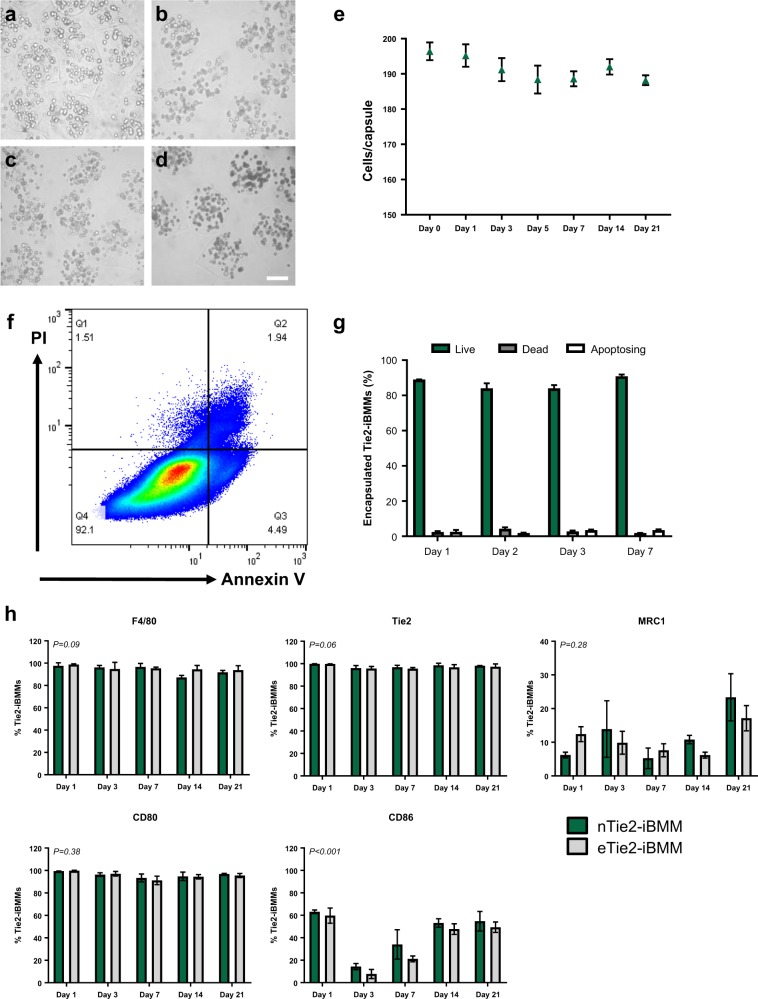
The effect of prolonged encapsulation on Tie2-iBMMs. **a-d** Alginate capsules seeded with Tie2-iBMMs at days: **a** 1, **b** 7, **c** 14 and **d** 21 postencapsulation. **e** Tie2-iBMM retention within the capsules was quantified up to day 21 following encapsulation (*n* = 5/group, *P* = n/s by one-way ANOVA). **f** Flow cytometric analysis of cell viability using annexin V/PI staining (Q1 = cell debris; Q2 = dead cells; Q3 = apoptosing cells; Q4 = live cells). **g** Quantification of annexin V/PI staining of encapsulated Tie2-iBMMs at days 1, 2, 3 and 7 post-encapsulation (*n* = 4/group, *P* = n/s by two-way ANOVA and Bonferroni post-test). **h** Measurement of cell phenotype by flow cytometry in nTie2-iBMMs (green) and eTie2-iBMMs (grey) up to 21 days in vitro (*n* = 5/group, *P* = *n/s P* < 0.001 by two-way ANOVA and *P* = *n/s* Bonferroni post-test). Error bars = s.d. Scale bar = 100 µm

Encapsulated Tie2-iBMMs (eTie2-iBMMs) were assessed longitudinally, in vitro, for cell viability and phenotype in order to ascertain whether encapsulation was detrimental to cell health (Fig. 2, Table 1). Microscopic analysis of the capsules demonstrated maintenance of capsule integrity and retention of the cells within the capsules up to day 21 postencapsulation (Fig. 2a–d). There was no significant loss of cells from the capsules in vitro up to day 21 (Fig. 2e, day 0: 196 ± 2.5 vs. day 21: 188 ± 1.4 cells/capsule). Analysis of cell viability by annexin V and propidium iodide (PI) staining (Fig. 2f, g, Table 1), across 7 days in vitro, demonstrated that the majority of cells remain viable.

**Table 1:** Assessment of encapsulated Tie2-iBMM viability in culture

		Day 1	Day 2	Day 3	Day 7	*P* value
**Live**		89.00 ± 0.1	84.03 ± 2.9	84.03 ± 1.8	90.85 ± 1.1	n/s
**Dead**		2.43 ± 0.5	4.28 ± 8.4	2.78 ± 0.5	1.90 ± 0.1	n/s
**Apoptosing**		2.52 ± 1.1	1.90 ± 0.3	3.57 ± 0.3	3.57 ± 0.4	n/s

We assessed the phenotype of macrophages following long-term in vitro culture of Tie2-iBMMs in alginate capsules. After 21 days, the expression of Tie2 and the mouse macrophage marker, F4/80, on naked Tie2-iBMMs (nTie2-iBMMs) remained constant and was comparable to that of eTie2-iBMMs (Fig. 2h, Supplementary Data, Table S1). Expression of the ‘M1’ macrophage markers CD80 and CD86 did not differ between nTie2-iBMMs and eTie2-iBMMs at any time point (Fig. 2h, Supplementary Data, Table S1). Expression of Mannose receptor C-type 1 (MRC1), an ‘M2’ macrophage marker, was not significantly different between nTie2-iBMMs and eTie2-iBMMs at any time point (Fig. 2h, Supplementary Data, Table S1).

### The effect of alginate encapsulation on Tie2-iBMM function

**Fig. 3:**
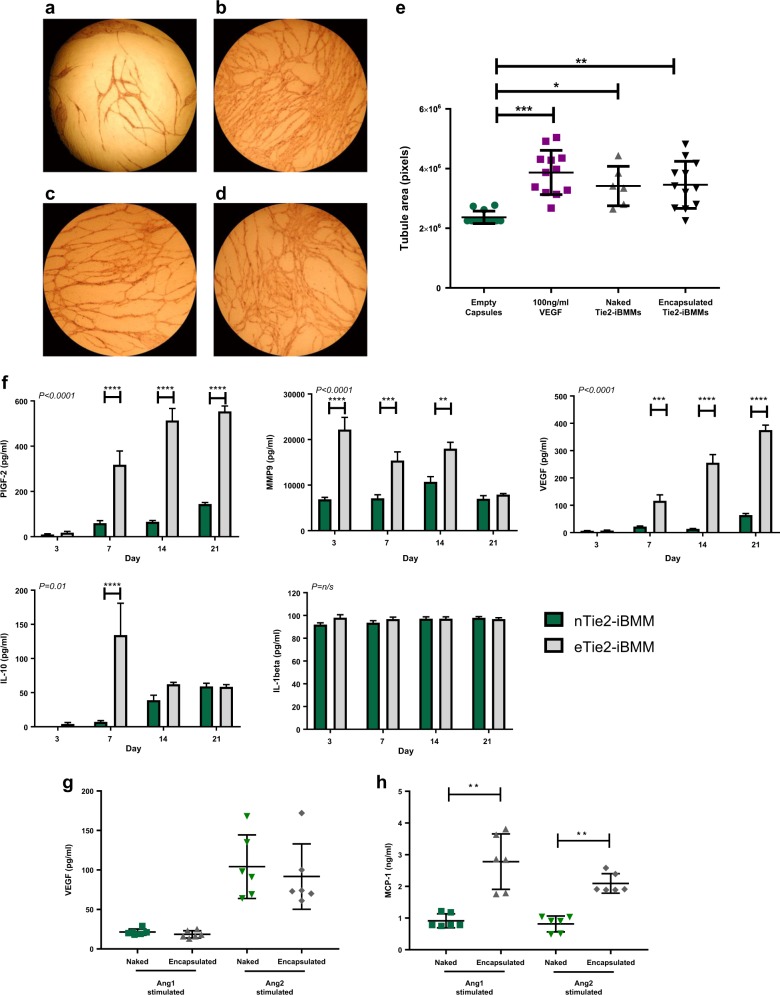
The effect of alginate encapsulation on the pro-angiogenic function of Tie2-iBMMs. **a–d** Representative light microscope images of HUVEC/fibroblast angiogenesis assays for HUVECs co-cultured with empty alginate capsules **a**, 100 ng/ml VEGF **b**, naked Tie2-iBMMs **c** or alginate encapsulated Tie2-iBMMs **d**. **e** HUVEC tubule area was compared (*n* = 6–12/group, *P = 0.05 **P = 0.01 ***P = 0.0001 by Kruskal Wallis test, error bars = s.d.). **f** Quantification of PlGF-2, VEGF, MMP9, IL-10 and IL-1β secreted by nTie2-iBMMs (green) and eTie2-iBMMs (grey) following in vitro culture for up to 21 days (*n* = 5/group, **P = 0.01, ***P = 0.001, ****P < 0.001 by two-way ANOVA and Bonferroni post-test, error bars = s.e.m.). **g**, **h** Quantification of Vegfa164 **g** and MCP-1 **h** secreted by stimulated Tie2-iBMMs using ELISA (*n* = 6/group, **P = 0.01 by Mann Whitney test, error bars = s.d.)

Quantification of human umbilical vein endothelial cell (HUVEC) tubule formation induced by culturing in the presence of either empty capsules (Fig. 3a), vascular endothelial growth factor (Vegfa, Fig. 3b), nTie2-iBMMs (Fig. 3c) or eTie2-iBMMs (Fig. 3d) demonstrated increased EC tubule area in eTie2-iBMM co-cultures compared with empty capsules (Fig. 3e, *P* = 0.002), and this was comparable to that induced by the Vegfa positive control (*P* > 0.1) and nTie2-iBMMs (*P* > 0.9).

In order to determine whether encapsulation of Tie2-iBMMs affected the secretion of factors that may promote or inhibit angiogenesis, we compared the conditioned media produced by nTie2-iBMMs and eTie2-iBMMs left in culture up to 21 days. We found a significantly higher expression of Placenta growth factor-2 (PlGF-2) and Vegfa 7, 14 and 21 days following encapsulation of Tie2-iBMMs compared with non-encapsulated cells (Fig. 3f, Supplementary Data, Table S2). Expression of Matrix metalloproteinase-9 (MMP9) was significantly higher in eTie2-iBMMs compared with nTie2-iBMMs at days 3, 7 and 14 post-encapsulation (Fig. 3f, Supplementary Data, Table S2). Secretion of the pro-inflammatory cytokine Interleukin-1β (IL-1β) was not affected by encapsulation, however, expression of the anti-inflammatory cytokine Interleukin-10 (IL-10) was significantly greater at day 7 following encapsulation (Fig. 3f, Supplementary Data, Table S2).

We sought to establish whether cell encapsulation hindered the signalling of pro-angio-/arteriogenic cells via soluble ligand binding (angiopoietins), as the cytokine milieu in the ischaemic limb is thought to modulate injected therapeutic cells in this manner.^20^ Moreover, for encapsulated cells to exert their beneficial effect, the biomaterial used must not deleteriously affect the secretion of soluble factors that may promote tissue regeneration in a paracrine fashion. TIE2 receptor phosphorylation can be induced by both Ang-1 and Ang-2, although there is debate as to which ligand induces the most potent angiogenic response in TEMs.^20^ The secretion of Vegfa by Tie2-iBMMs stimulated with Ang-1 and Ang-2 was not different between naked and encapsulated cells (Fig. 3g). As well as assessing the effect of encapsulation on the paracrine function of Tie2-iBMMs, we sought to investigate whether production of chemokines involved in monocyte recruitment to the ischaemic limb was affected. Monocyte chemoattractant protein-1 (MCP-1) promotes the recruitment of monocytes to the ischaemic limb, which subsequently differentiate into M2 macrophages and enhance arteriogenesis.^21–23^ MCP-1 production was greater in eTie2-iBMMs compared with nTie2-iBMMs following stimulation by both Ang-1 and Ang-2 (Fig. 3h, *P* < 0.01 for both), indicating that eTie2-iBMMs may act not only through enhanced paracrine function, but also through recruitment of cells implicated in driving a pro-arteriogenic response.

**Fig. 4:**
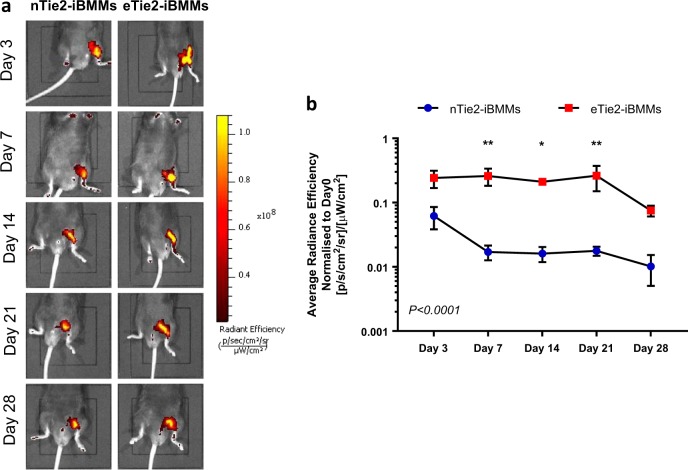
The effect of alginate encapsulation on cell retention within the ischaemic murine hindlimb. **a** Tie2-iBMMs stained with VivoTrack680 biofluorescent dye were either directly injected (nTie2-iBMMs) into the ischaemic limb of mice or encapsulated within alginate (eTie2-iBMMs), and their retention tracked using an IVIS Spectrum In Vivo Imaging System over 28 days. **b** The average radiance efficiency at each time point was normalised to day 0 and compared between treatment groups (*n* = 4/group, *P* < 0.0001 by two-way ANOVA *P = 0.05 **P = 0.01 by Bonferroni post-test, error bars = s.e.m.)

Given that in vitro culture demonstrated Tie2-iBMM viability and phenotype could be maintained after prolonged encapsulation within alginate capsules, we assessed whether eTie2-iBMMs were better retained following delivery into the murine ischaemic hindlimb compared with naked cells. We found that although there was a reduction in biofluorescence in both treatment groups over 28 days (Fig. 4a), eTie2-iBMMs were significantly better retained at days 7, 14 and 21 than nTie2-iBMMs (Fig. 4b, *P* < 0.0001).

**Fig. 5:**
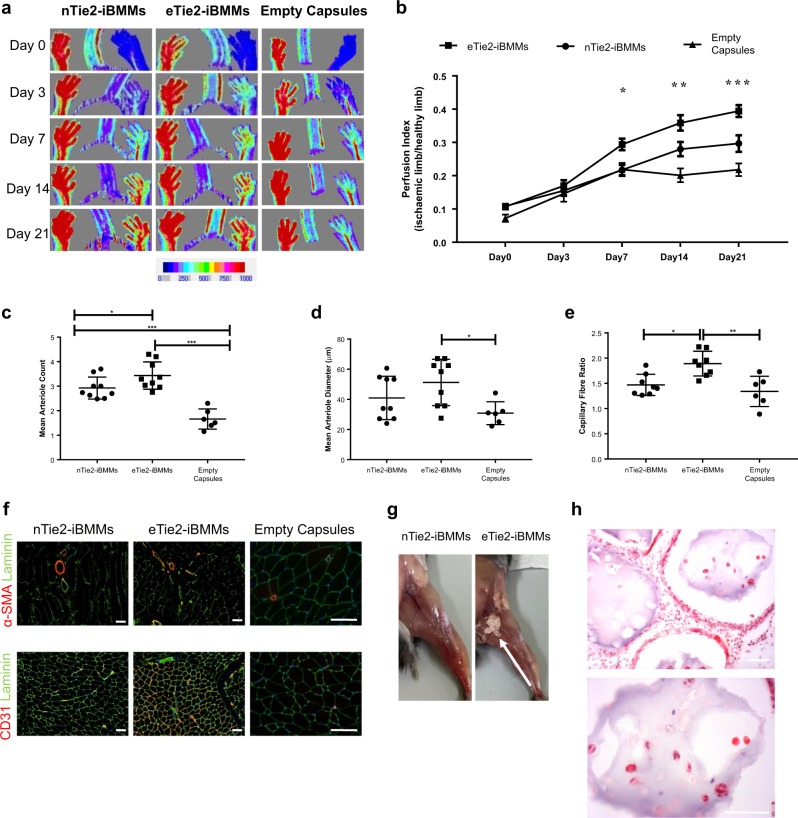
The effect of cell encapsulation on revascularisation of the murine ischaemic hindlimb. **a** Laser Doppler images of mice treated with direct injection of naked or encapsulated Tie2-iBMMs, or acellular alginate capsules, measured over 21 days (*n* = 11–15/group). **b** Perfusion index of murine hindlimbs following induction of ischaemia up to day 21 (ischaemic limb flux/contralateral limb flux, *P* < 0.05 by two-way ANOVA *P = 0.05 **P = 0.01 ***P = 0.0001 by Bonferroni post-test, error bars = s.e.m.). **c**, **d** Mean α-SMA^+^ arteriole number per field of view **c** and diameter **d** in the adductor muscle of mice following HLI surgery at day 21 (*n* = 6–9/group, *P = 0.05 ***P = 0.001 by Kruskal Wallis test, error bars = s.d.). **e** Capillary fibre:ratio of gastrocnemius muscle samples harvested from mice at day 21 (CD31^+^ capillaries:laminin^+^ muscle fibres, *n* = 6–9/group, *P = 0.05 **P = 0.01 by Kruskal Wallis test, error bars = s.d.). **f** Representative fluorescence microscopy images of arteriole staining for α-SMA (red) and laminin (green) and capillary/fibre staining for CD31 (red) and laminin (green). **g** Murine hindlimbs at day 21 treated with direct injection of naked or encapsulated cells (white arrow). **h** H&E analysis of capsules harvested from HLI mice at day 21. Scale bar = 100 µm

Encapsulated Tie2-iBMMs induced greater reperfusion of ischaemic hind limbs than treatment with nTie2-iBMMs (*P* < 0.01). Mice injected with eTie2-iBMMs or nTie2-iBMMs demonstrated greater revascularisation of the ischaemic limb over 21 days compared with animals treated with empty alginate capsules (Fig. 5a, b, *P* < 0.0001 and *P* < 0.05, respectively). Histological analysis revealed an increase in the number of arterioles, (Fig. 5c, f, *P* = 0.03), and a trend to increased arteriole diameter (Fig. 5d, f, *P* = 0.057) of α-smooth muscle actin (α-SMA^+^) arterioles; as well as increased angiogenesis (capillary:fibre ratio, Fig. 5e, f, *P* = 0.023) in ischaemic muscle specimens of mice treated with eTie2-iBMMs compared with nTie2-iBMMs. Mice treated with empty alginate capsules had significantly less angiogenesis and arteriogenesis compared with those treated with eTie2-iBMMs (Fig. 5c–e, *P* = 0.01). Alginate capsules persisted in the hindlimb after 21 days (Fig. 5g), and still contained cells at this time (Fig. 5h).

**Fig. 6:**
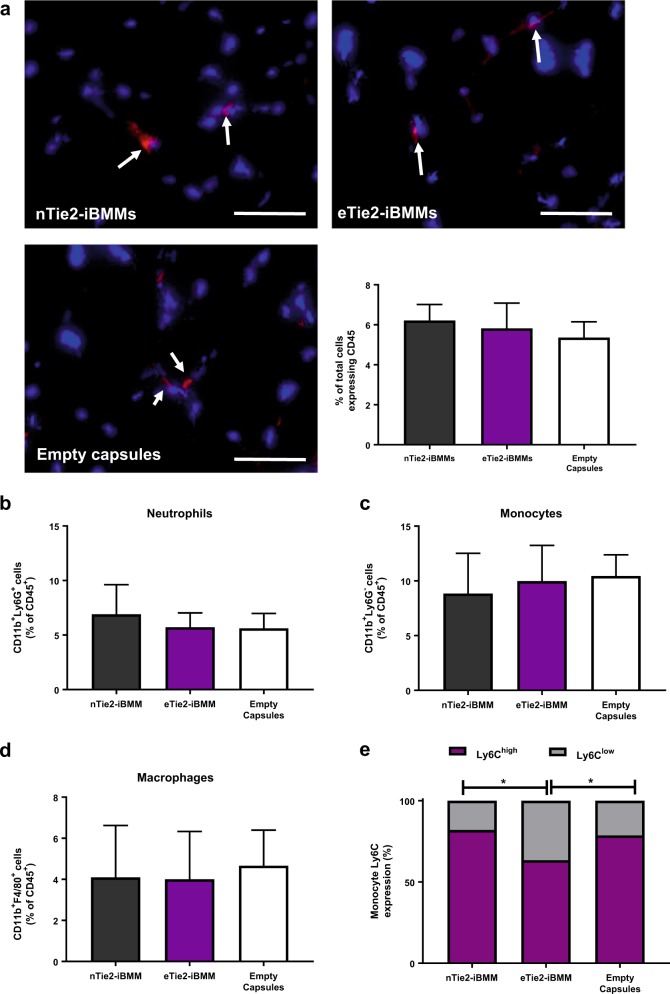

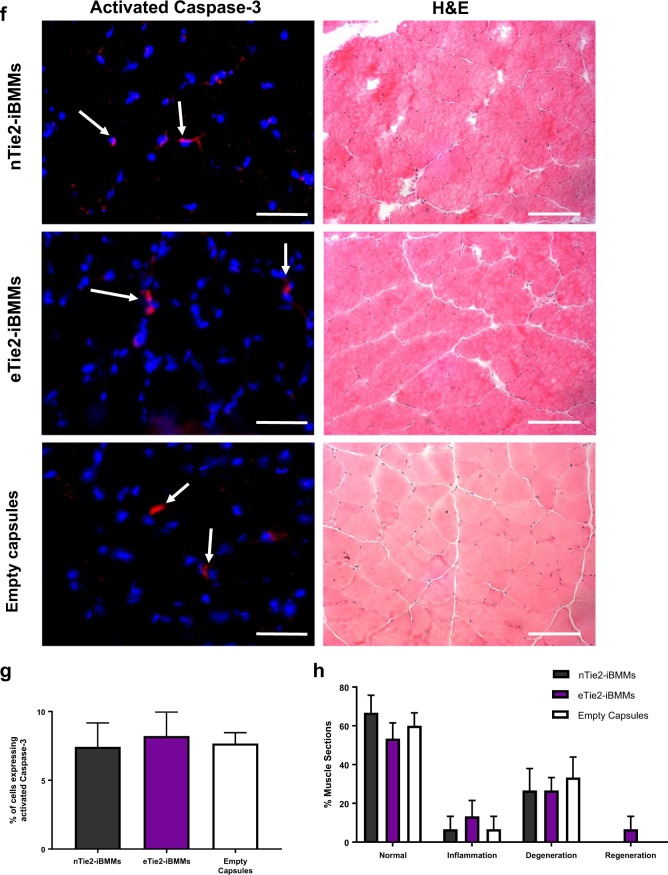
The effect of eTie2-iBMM treatment of the ischaemic hindlimb on inflammation, apoptosis and muscle damage. **a** CD45^+^ cells (white arrow) within the adductor muscle of nTie2-iBMM, eTie2-iBMM or empty capsule-treated mice. CD45^+^ cells were quantified as a total proportion of DAPI-stained cells (*n* = 5/group, *P* = *n/s* by Kruskal Wallis test, error bars = s.e.m.). **b–d** Quantification of **b** neutrophil, **c** monocyte and **d** macrophage content of ischaemic hindlimb muscle after 7 days following delivery of nTie2-iBMMs (grey), eTie2-iBMMs (purple) or empty alginate capsules (white). Data are represented as a proportion of CD45^+^ cells (*n* = 5/group, *P* = *n/s* by Kruskal Wallis test, error bars = s.d.). **e** Analysis of proportion of Ly6C^high^ (purple) and Ly6C^low^ (grey) monocytes isolated from ischaemic muscle (*n* = 5/group, *P* = 0.05 by Kruskal Wallis test). **f** Representative fluorescence microscopy images of cells (blue, DAPI) expressing activated caspase-3 (red, white arrow) in adductor muscle specimens of ischaemic adductor muscle fibres from nTie2-iBMM, eTie2-iBMM and empty alginate capsule-treated mice; and H&E stained microscopy images of adductor muscle from nTie2-iBMM, eTie2-iBMM and empty alginate capsule-treated mice. Quantification of **g** cells expressing activated caspase-3 (*n* = 4/group, *P* = *n/s* by Kruskal Wallis test, error bars = s.e.m.) and **h** muscle damage/repair (*n* = 5/group, *P* = *n/s* by Kruskal Wallis test, error bars = s.e.m.) in ischaemic adductor muscle from mice treated with nTie2-iBMMs, eTie2-iBMMs and empty alginate capsules. Scale bars = 100 µm

There was no significant difference in the number of CD45^+^ cells in hindlimbs injected with eTie2-iBMMs compared with nTie2-iBMMs (Fig. 6a). Deep phenotyping of the CD45^+^ population showed no significant difference in the proportion of neutrophils (CD11b^+^Ly6G^+^), or monocytes and macrophages (CD11b^+^Ly6G^−^ and CD11b^+^F4/80^+^ cells, Fig. 6b–d, Supplementary Data, Table S3). Treatment with eTie2-iBMMs was associated with a significantly reduced proportion of the endogenous CD11b^+^Ly6G^−^ monocytes expressing Ly6C (Ly6C^high^, Fig. 6e, Supplementary Data, Table S3) compared with nTie2-iBMM and empty capsule-treated mice (*P* < 0.05). Histological analysis of muscle specimens revealed no significant difference in the number of cells expressing the apoptosis marker activated caspase-3 between treatment groups (Fig. 6f, g), or any difference in muscle damage between treatment groups (Fig. 6f, h).

## Discussion

To date, cell-based therapies for the treatment of CLI have demonstrated limited efficacy in clinical trials.^4–6^ A possible contributing factor to these modest results is poor cell retention following direct injection of cells into the ischaemic limb. This suggests a need for an alternative delivery system, such as encapsulation of therapeutic cells within a biocompatible material prior to implantation that promotes cell retention to ensure a better outcome.

This study investigates the effect of alginate encapsulation on the phenotype and function of a pro-angio/arteriogenic murine macrophage line (Tie2-iBMMs), in revascularising the ischaemic limb. We describe a GMP-compliant methodology for the consistent generation of uniform alginate capsules containing these cells that does not adversely affect their viability, phenotype and function in vitro. Encapsulation enhanced Tie2-iBMM retention following implantation into the ischaemic hindlimb and this was associated with significantly greater angio/arteriogenesis and overall limb revascularisation compared with non-encapsulated Tie2-iBMMs.

Tie2-expressing macrophages are thought to facilitate revascularisation either through a paracrine action^24,25^ or via direct contact with ECs^26^ and, therefore, their utility as therapeutic cells necessitates their delivery in close proximity to an ischaemic region to maximise their revascularisation potential.^27^ Maintenance of their retention at the site of delivery is thought to be another important factor in achieving optimal therapeutic benefit, with significant cell loss from the site of implantation noted when directly injected into both the ischaemic heart and limb.^12,28^ Cell encapsulation maintains retention and has proved efficacious in different clinical settings, including pancreatic islet cell and hepatocyte transplantation for the treatment of diabetes and liver failure.^29,30^ The data presented demonstrates that Tie2-expressing macrophage secretion of pro-angio/arteriogenic cytokines is preserved or even enhanced following encapsulation. PlGF-2, VEGF and MMP9 have proven potential for promoting ischaemic tissue repair through induction of angiogenesis, progenitor cell recruitment and improved integration of injected cellular biomaterials and, therefore, the greater degree of limb reperfusion in eTie2-iBMM-treated animals could be attributed to the improved retention of these cells in the ischaemic region, facilitating the action of these growth factors.^31–33^ In addition to providing a physical barrier for preventing cell loss through wash out by the vascular and lymphatic systems, alginate encapsulation of cells has also been shown to inhibit migration of cells out of the capsule into the surrounding host tissues.^15^

An advantage of encapsulating cells, in addition to improving retention, is their immuneprivileged status within the capsule.^34^ Although immunogenicity is not a consideration when using autologous cells for therapeutic purposes, murine studies suggest that co-morbidities associated with CLI can adversely affect the angio/arteriogenic potential of monocyte/macrophages.^35^ Allogeneic macrophages from healthy individuals, that may have more potent angio/arteriogenic properties for promoting limb salvage, could be used in combination with encapsulation technologies, to enhance the efficacy of cell-based strategies. The protection from host immunity conferred by encapsulation of cells from allogeneic sources, warrants further investigation in the context of ischaemia. CLI patients frequently suffer with multiple co-morbidities, and the functional potency of their cells should be compared with those isolated from healthy subjects in order to determine the most suitable source of cells for successful therapy.

The present study highlights the promise offered, through the use of a GMP-compliant biomaterial encapsulation process, to enhance the efficacy of cell therapies for treating limb ischaemia. We employed the murine macrophage iBMM cell line in our experiments to ensure replicability and fair comparison in our proof of concept study. Further studies, using human macrophages in place of the mouse cell line tested here, would be required to allow the translation of this work into clinical trials. Here, we show not only an improvement in the method for delivering cells, but also the potential for a whole new cell product for therapeutic use when human macrophages are encapsulated under GMP conditions. Sodium alginate is a well-established material for the purposes of cell encapsulation, although there now exists an expansive range of biomaterials that have been engineered to specifically promote the reparative function of cells contained therein.^36–38^ Biomaterial-based cell therapies may be further enhanced through engineering to allow for the temporal release of pro-angio/arteriogenic factors that may increase the potency of encapsulated cells. Growth factor-containing hydrogel cores within alginate microcapsules are postulated to improve cell survival,^39^ with MSC-VEGF co-encapsulation demonstrating promise in the treatment of myocardial infarction.^40^ Co-encapsulation of different cells may also enhance therapeutic cell function and survival.^41,42^ It is possible therefore, that although the present study highlights the benefit of cellular encapsulation in promoting retention of therapeutic cells and their activity in revascularising the ischaemic limb, there could be scope for further improvements to enhance their efficacy through the development of co-encapsulation modalities.

In summary, these studies provide an optimised methodology for the generation of alginate capsules containing pro-angio/arteriogenic macrophages, and show that encapsulation in this biopolymer is not detrimental to cell viability, phenotype or function. These data show that encapsulation both enhances macrophage retention and their pro-angiogenic/arteriogenic potential in the ischaemic murine hindlimb, which leads to greater limb perfusion, compared with naked cells. This work may have important implications for cell-based therapies currently being trialled for treatment of CLI.

## Methods

### Cell culture

Murine bone marrow-derived macrophages were immortalised using a lentiviral vector containing the SV40 large T Antigen coding sequence to form iBMMs.^43^ Vesicular stomatitis virus-pseudotyped, third generation lentiviruses were produced by plasmid transfection of 293T cells. The SV40 large T antigen coding sequence was cloned into the SFFV promoter-containing lentivirus using BamHI and SaII restriction enzymes, and the resultant lentivirus used for transduction. Tie2 expression was subsequently induced via a second lentiviral transduction.^43,44^ Tie2-iBMMs were cultured in complete medium (IMDM (Gibco, UK), 20% foetal calf serum containing 2 mM glutamine, 1% (v/v) antibiotic/antimycotic and 50 ng/ml macrophage colony stimulating factor (M-CSF, Peprotech, UK)) under standard conditions (37 °C, 21% O_2_, 5% CO_2_).

### Encapsulation of Tie2-iBMMs

SLG20 alginate (1.5% (w/v), Pronova Biomedical) was prepared in 0.9% (w/v) NaCl, and cells resuspended in alginate at a concentration of 1.0 × 10^7^ cells/ml. Capsules were generated using a GMP-compliant BUCHI B-395 Pro encapsulator, set at a flow rate of 12.0 ml/min, with the cell solution passing through a 120 µm nozzle vibrating at 1800 Hz, and a 6.8 kV electric field, into a polymerisation solution (1.2% (w/v) CaCl2, 0.9% (w/v) NaCl, Tween-20). Capsules were subsequently washed in 0.9% (w/v) NaCl. Capsule diameter and cell number/capsule was determined by counting the number of cells within ten capsules from three separate experiments under a brightfield microscope. The GMP-grade encapsulation system generated sterile capsules that contained the murine macrophage cell line in order to minimise the possibility of infection and hence any inflammation that might confound our revascularisation results in our animal hindlimb ischaemia (HLI) model.

### Digestion of alginate capsules

Capsules were centrifuged to remove media (300*g*, 5 min) and resuspended in chelation solution (30 mM EDTA in 55 mM sodium citrate), prior to 5 min incubation at 37 °C with regular vortexing. The digestion solution was passed through a 70 µm cell strainer to remove undigested alginate. Cells from the digested capsules were washed and centrifuged at 300g for 5 min to pellet.

### Preparation of single-cell suspensions from ischaemic muscle for flow cytometry

Adductor muscle samples were harvested from treated animals 7 days after the procedure. Briefly, cells were isolated from dissected tissue following 30 min incubation in a tissue digestion buffer (0.5% bovine serum albumin, 1 mg/ml collagenase, 1 mM EDTA, 500 units/ml hyaluronidase and 100 units/ml DNase I in dPBS (Sigma)). Filtered tissue digests were subject to red blood cell lysis (BD Bioscience) and washed prior to staining and analysis using flow cytometry.

### Flow cytometry

Cell viability and phenotype were assessed using either a MACSQuant (Miltenyi Biotec, UK) or AttuneNxT (Thermo Scientific, UK) flow cytometer. Cells were harvested from (i) monolayer culture (naked—nTie2-iBMMs) and alginate capsules (encapsulated—eTie2-iBMMs) at days 1, 3, 5, 7, 14 and 21 post-encapsulation and (ii) digested adductor muscle specimens. Cells were washed and FcR receptors blocked using FcR blocking reagent (Miltenyi Biotec, UK). Cell viability was determined using a Live-Dead Staining Kit (Thermo Fisher Scientific, UK) for annexin V and PI according to manufacturer’s instructions. Antibodies for assessment of cell phenotype or muscle cell content are listed in Supplementary Data, Table S4. All experiments utilised fluorescence minus one controls to determine positive cell surface expression, and analysis of acquired data was carried out using FlowJo V10 software. Gating panels are detailed in Supplementary Data, Figures S5 and S6.

### In vitro angiogenesis assay

The angiogenic potential of eTie2-iBMMs was assessed using a previously described HUVEC/fibroblast co-culture assay,^45^ and compared with HUVEC tubule formation induced by empty alginate capsules. Media containing 100 ng/ml VEGF was used as a positive control. HUVEC tubule formation was quantified after 14 days using ImageProPlus software.

### Luminex quantification of secreted cytokines

A custom Luminex assay (R&D Systems, UK) for murine PlGF-2, VEGF, MMP9, IL-1β and IL-10 was used to quantify secreted protein levels in conditioned media collected from nTie2-iBMM and eTie2-iBMM cell cultures at days 3, 7, 14 and 21. The assay was carried out according to manufacturer’s instructions, and data captured using a Bio-Plex MAGPIX system (BioRad, UK).

### Vegfa and MCP-1 ELISA

The secretion of Vegfa and MCP-1 by Ang-1/Ang-2-stimulated Tie2-iBMMs was assessed using ELISA (R&D Systems, UK) according to manufacturer’s instructions. Briefly, either nTie2-iBMMs or eTie2-iBMMs were stimulated with 200ng/ml Ang-1 or Ang-2 for 24 h. Media was then replaced with serum-free iBMM media for a further 24 h and conditioned media subsequently collected for analysis.

### Animal source and husbandry

This study complied with ethical regulations stipulated by U.K. Animals (Scientific Procedures) Act, 1986 and associated guidelines, and the study protocol approved by the Home Office. Male C57BL/6 mice aged 8–10 weeks were procured from Charles River Laboratories. All animals were randomised prior to experimentation and during acquisition of data, observers were blinded to these allocations. Animals were maintained in individually ventilated cages, and their health status monitored throughout the course of the experiment.

### In vivo biofluorescence

Tie2-iBMMs were stained with VivoTrack680 biofluorescent dye (Perkin Elmer, UK) according to manufacturer’s instructions. Cells were then either directly injected into the adductor muscle of mice undergoing hindlimb ischaemia surgery, or encapsulated in alginate prior to implantation in operated mice. Each mouse received 1 × 10^6^ Tie2-iBMMs. Radiance efficiency was quantified using an IVIS Spectrum In Vivo imaging system (Perkin Elmer) at days 0, 3, 7, 14, 21 and 28 using a 60 s exposure time to assess changes in fluorescence intensity using Living Imaging v4.5 software.

### Murine model of HLI

Unilateral hindlimb ischaemia was surgically induced in 8-week-old C57BL/6 male mice (*n* = 15/group) by ligation of the femoral artery proximal and distal to the profunda femoris and excision of the intervening segment. nTie2-iBMMs were either directly injected into the adductor muscle or encapsulated and layered onto the muscle. Empty alginate capsules were layered onto the muscle as a control. Paw perfusion was quantified by laser Doppler perfusion imaging (LDPI, Moor Instruments, UK) at 3, 7, 14 and 21 days. Adductor and gastrocnemius muscles were harvested at day 21 for histological analysis.

### Histological analysis

Muscle specimens were fixed in 4% paraformaldehyde and dehydrated in increasing concentrations of sucrose (15, 30 and 40%) prior to snap-freezing in isopentane. Five consecutive 10 μm sections were stained from three areas of each muscle specimen (500 μm separation), and analysed for measures of either arterio- or angiogenesis. Arteriogenesis was measured in adductor muscle specimens by staining for α-SMA and laminin; whilst angiogenesis, in gastrocnemius muscle, was measured by quantification of capillary:fibre ratio using antibodies against CD31-PECAM and laminin. The number of CD45^+^ cells per field of view was quantified in adductor muscle specimens and cell apoptosis quantified by staining for activated Caspase-3. Antibody information is listed in Supplementary Table S4. Cell retention within implanted capsules, harvested from the operated limb at day 21, was analysed using H&E stain. Muscle fibre damage in the ischaemic limb was assessed by H&E staining of ischaemic adductor muscle sections, with fibres characterised as normal, damaged or regenerating using a standard protocol.^46^ Fluorescent and histological staining was assessed with a Nikon Ti Eclipse microscope using NIS-Elements BR microscopy software.

### Statistical analysis

All statistical analysis was performed using GraphPad Prism 7 software. Technical and experimental repeats were conducted to ensure that experiments were powered to at least 80%. Statistical significance was analysed by one- or two-way ANOVA and appropriate post-hoc tests, or by Mann Whitney/Kruskal Wallis test, as specified in the figure legends. A threshold of *P* < 0.05 was defined as statistically significant. Data are presented as mean ± SD.

### Reporting summary

Further information on experimental design is available in the Nature Research Reporting Summary linked to this article.

## Supplementary information

Supplemental Material

Reporting Summary

## Data Availability

The data that supports the findings of this study are available from the corresponding author upon reasonable request.

## Supplementary information

**Supplementary information** accompanies the paper on the *npj Regenerative Medicine* website (10.1038/s41536-019-0068-5).
