# Supplementary material for: Encapsulation of macrophages enhances their retention and angiogenic potential
Source: NPJ Regen Med. 2019 Mar 20;4:6. doi: 10.1038/s41536-019-0068-5 (PMC6426993; doi:10.1038/s41536-019-0068-5)
Supplement: Supplementary file 1 — Supplemental Material [file 41536_2019_68_MOESM1_ESM.docx]

**Supplementary Table S1** Assessment of encapsulated Tie2-iBMM phenotype in culture

|  |  | **Day 1** | **Day 3** | **Day 7** | **Day 14** | **Day 21** | ***P* value** |
| --- | --- | --- | --- | --- | --- | --- | --- |
| **Tie2** | nTie2-iBMM | 99.9±0.1 | 96.1±2.3 | 96.8±1.7 | 98.6±0.2 | 98.1±0.2 | n/s |
|  | eTie2-iBMM | 99.8±0.1 | 95.6±1.9 | 95.6±1.0 | 96.7±2.4 | 97.3±2.5 |  |
| **F4/80** | nTie2-iBMM | 97.7±2.7 | 96.1±1.8 | 96.6±3.1 | 87.3±1.7 | 91.8±1.8 | n/s |
|  | eTie2-iBMM | 98.7±0.8 | 94.8±6.0 | 95.4±1.0 | 94.4±3.5 | 93.8±4.0 |  |
| **MRC1** | nTie2-iBMM | 6.20±0.8 | 13.9±8.4 | 5.24±3.1 | 10.8±1.2 | 23.4±7.0 | n/s |
|  | eTie2-iBMM | 12.4±2.2 | 9.86±3.4 | 7.62±1.9 | 6.18±0.9 | 17.1±3.8 |  |
| **CD80** | nTie2-iBMM | 99.5±0.1 | 96.3±1.6 | 93.4±3.5 | 94.8±3.7 | 96.8±0.6 | n/s |
|  | eTie2-iBMM | 99.7±0.5 | 97.1±2.0 | 91.2±3.8 | 94.4±1.9 | 95.5±1.8 |  |
| **CD86** | nTie2-iBMM | 63.1±1.6 | 14.3±2.8 | 34.0±13 | 53.2±3.8 | 54.8±8.7 | n/s |
|  | eTie2-iBMM | 59.7±6.7 | 7.7±4.1 | 21.2±2.6 | 47.7±4.7 | 49.4±4.6 |  |

**Supplementary Table S2** Soluble PlGF-2, VEGF, MMP9, IL-1β and IL-10 production by nTie2-iBMM and eTie2-iBMM *in vitro*

|  |  | **Day 3** | **Day 7** | **Day 14** | **Day 21** |
| --- | --- | --- | --- | --- | --- |
| **PlGF-2**  **(pg/ml)** | nTie2-iBMM | 9.96±6.6 | 59.6±26 | 65.6±0.13.9 | 144±15.6 |
|  | eTie2-iBMM | 17.9±12 | 317±136**** | 513±120**** | 554±53.2**** |
| **VEGF**  **(pg/ml)** | nTie2-iBMM | 6.63±1.9 | 21.9±5.4 | 13.5±4.1 | 64.1±12 |
|  | eTie2-iBMM | 7.34±4.6 | 116±49*** | 256±67**** | 375±41**** |
| **MMP9**  **(ng/ml)** | nTie2-iBMM | 6.87±0.9 | 7.09±1.8 | 10.7±2.6 | 6.98±1.6 |
|  | eTie2-iBMM | 22.2±5.9**** | 15.4±4.3*** | 18.0±3.1** | 7.91±0.5 |
| **IL-1β**  **(pg/ml)** | nTie2-iBMM | 91.9±1.6 | 93.7±1.7 | 97.1±1.7 | 97.9±1.1 |
|  | eTie2-iBMM | 98.0±2.6 | 96.8±1.7 | 97.1±1.7 | 96.8±1.2 |
| **IL-10**  **(pg/ml)** | nTie2-iBMM | 0.00±0.0 | 7.12±1.9 | 38.9±7.3 | 59.1±4.6 |
|  | eTie2-iBMM | 4.18±2.1 | 134.3±47 | 62.2±2.8** | 58.4±3.3 |

***P<0.01 ***P<0.001 ****P<0.0001*

**Supplementary Table S3** Assessment of ischaemic hindlimb inflammatory cells following nTie2/eTie2-iBMM treatment

|  | **nTie2-iBMMs** | **eTie2-iBMMs** | **Empty Capsules** | ***P* value** |
| --- | --- | --- | --- | --- |
| (%) Neutrophils  (%CD45^+^ cells) | 6.90±2.7 | 5.72±1.3 | 5.62±1.4 | n/s |
| (%) Monocytes  (%CD45^+^ cells) | 8.84±3.7 | 9.98±3.3 | 10.4±1.9 | n/s |
| (%) Macrophages  (%CD45^+^ cells) | 4.10±2.5 | 4.00±2.3 | 4.66±1.7 | n/s |
| Monocytes: Ly6C^high^  Monocytes: Ly6C^low^ | 82.0±8.1  18.0±8.1 | 63.4±12  36.6±12 | 78.7±7.9  21.3±7.9 | **P*<0.05 |

*Kruskal-Wallis tests for nTie2-iBMMs vs eTie2-iBMMs and eTie2-iBMMs vs Empty capsules

**Supplementary Table S4** Antibody details

| **Antigen** | **Conjugate** | **Supplier** | **Assay Concentration** |
| --- | --- | --- | --- |
| CD3ε | PerCP-Cy5.5 | BD Biosciences  #551163 | 5µg/ml |
| CD11b | APCVio770 | Miltenyi Biotec  #130-113-232 | 1.5µg/ml |
| CD19 | PerCPVio700 | Miltenyi Biotec  #130-102-237 | 1.5µg/ml |
| CD45 | VioBlue | Miltenyi Biotec  #130-110-664 | 7.5µg/ml |
| CD80 | APCVio770 | Miltenyi Biotec  #130-116-399 | 7.5µg/ml |
| CD86 | VioBlue | Miltenyi Biotec  #130-102-438 | 1.5µg/ml |
| F4/80 | PEVio770 | Miltenyi Biotec  #130-142-901 | 7.5µg/ml |
| Ly6C | FITC | BD Biosciences  #553104 | 12.5µg/ml |
| Ly6G | AlexaFluor700 | Biolegend  #108422 | 12.5µg/ml |
| MRC1 | APC | Biolegend  #141708 | 5µg/ml |
| NK1.1 | PerCPVio700 | Miltenyi Biotec  #130-103-963 | 7.5µg/ml |
| Tie2 | PE | Biolegend  #124008 | 5µg/ml |
| α-SMA | Cy3 | Sigma Aldrich  #C6198 | 10µg/ml |
| Activated Caspase-3 | Unconjugated | Novus Biologicals  #AF835 | 15µg/ml |
| CD31-PECAM | Unconjugated | BD Pharminogen  #553370 | 5µg/ml |
| CD45 | Unconjugated | Abcam  #NB500-319 | 15µg/ml |
| Laminin | AlexaFluor488 | Novus Biologicals  #NB300-144G | 20µg/ml |


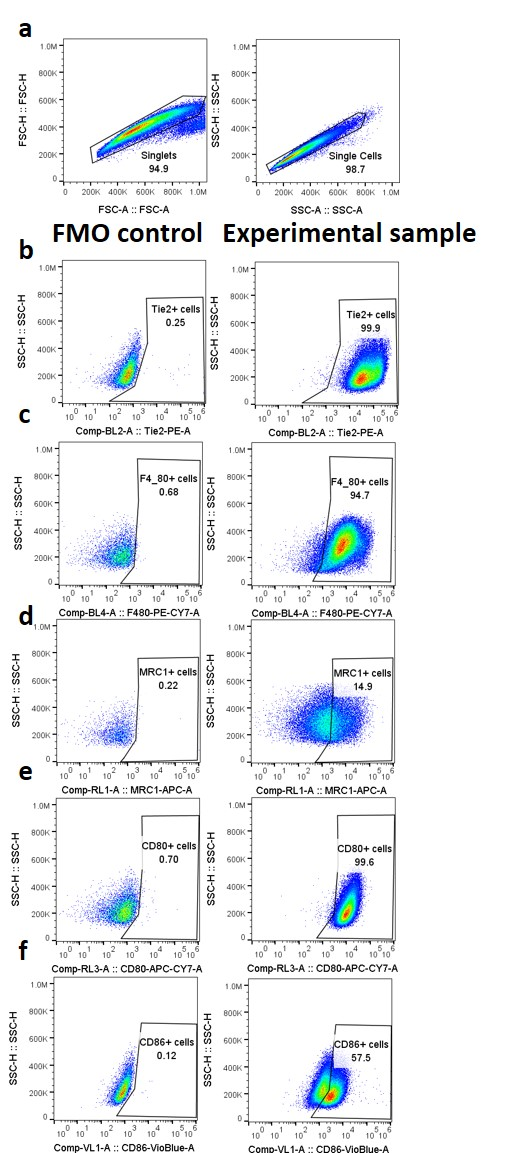


**Supplementary Figure S5** Flow cytometry gating strategy for longitudinal *in vitro* Tie2-iBMM phenotype analysis using fluorescence minus one (FMO) controls to define positive populations in experimental samples. (**a**) doublet cell exclusion using forward and side light scatter properties; (**b**) gating strategy for Tie2 expression; (**c**) gating strategy for F4/80 expression; (**d**) gating strategy for MRC1 expression; (**e**) gating strategy for CD80 expression; and (**f**) gating strategy for CD86 expression. These gating panels correspond to the data panels in Fig. 2h.


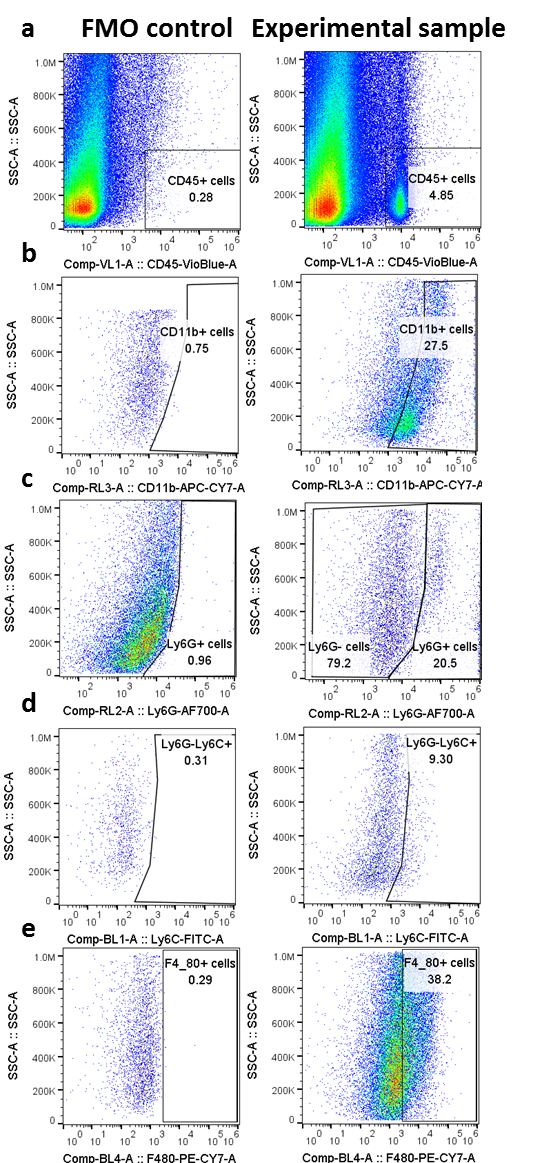


**Supplementary Data Figure S6** Flow cytometry gating strategy for analysis of ischaemic muscle inflammatory cell infiltrate using fluorescence minus one (FMO) controls to define positive populations in experimental samples. (**a**) gating strategy for CD45 expression; (**b**) gating strategy for CD11b expression; (**c**) gating strategy for Ly6G expression; (**d**) gating strategy for Ly6C expression; and (**e**) gating strategy for F4/80 expression. These gating panels correspond to the data panels in Fig. 6b-e.
